# Passing MRCP (UK) PACES: a cross-sectional study examining the performance of doctors by sex and country

**DOI:** 10.1186/s12909-018-1178-2

**Published:** 2018-04-06

**Authors:** Emily Unwin, Henry W. W. Potts, Jane Dacre, Andrew Elder, Katherine Woolf

**Affiliations:** 1 Research Department of Medical Education, University College London Medical School, Royal Free Hospital, GF 664, Rowland Hill Street, London, NW3 2PF UK; 2 Institute of Health Informatics, University College London, 222 Euston Road, London, NW1 2DA UK; 3 Royal College of Physicians, 11 St Andrews Place, London, NW1 4LE UK

**Keywords:** Clinical assessment, Examination performance, International medical graduates, MRCP, PACES, Sex difference

## Abstract

**Background:**

There is much discussion about the sex differences that exist in medical education. Research from the United Kingdom (UK) and United States has found female doctors earn less, and are less likely to be senior authors on academic papers, but female doctors are also less likely to be sanctioned, and have been found to perform better academically and clinically. It is also known that international medical graduates tend to perform more poorly academically compared to home-trained graduates in the UK, US, and Canada. It is uncertain whether the magnitude and direction of sex differences in doctors’ performance is variable by country. We explored the association between doctors’ sex and their performance at a large international high-stakes clinical examination: the Membership of the Royal Colleges of Physicians (UK) Practical Assessment of Clinical Examination Skills (PACES). We examined how sex differences varied by the country in which the doctor received their primary medical qualification, the country in which they took the PACES examination, and by the country in which they are registered to practise.

**Methods:**

Seven thousand six hundred seventy-one doctors attempted PACES between October 2010 and May 2013. We analysed sex differences in first time pass rates, controlling for ethnicity, in three groups: (i) UK medical graduates (*N* = 3574); (ii) non-UK medical graduates registered with the UK medical regulator, the General Medical Council (GMC), and thus likely to be working in the UK (*N* = 1067); and (iii) non-UK medical graduates without GMC registration and so legally unable to work or train in the UK (*N* = 2179).

**Results:**

Female doctors were statistically significantly more likely to pass at their first attempt in all three groups, with the greatest sex effect seen in non-UK medical graduates without GMC registration (OR = 1.99; 95% CI = 1.65-2.39; *P* < 0.0001) and the smallest in the UK graduates (OR = 1.18; 95% CI = 1.03-1.35; *P* = 0.02).

**Conclusions:**

As found in a previous format of this examination and in other clinical examinations, female doctors outperformed male doctors. Further work is required to explore why sex differences were greater in non-UK graduates, especially those without GMC registration, and to consider how examination performance may relate to performance in practice.

## Background

Sex differences among doctors are much discussed. On the one hand female doctors have poorer career outcomes compared to male doctors, for example earning less [1–3] and being less likely to be senior authors on academic papers [4, 5]. On the other hand, there is evidence that female doctors have better performance outcomes compared to male doctors. Female doctors are less likely to be subject to medico-legal action [6] even after controlling for specialty, time since qualification, and country of qualification [7]. A large study from the United States found that patients of female doctors had better clinical outcomes [8]. Academically a recent (currently unpublished) meta-analysis showed that female doctors tend to outperform men, with the largest effect in practical clinical examinations rather than in written examinations, which showed more gender parity.

Sex differences in doctors’ academic outcomes may be confounded by other variables such as ethnicity and country of qualification. Research from the UK, US, Netherlands, Canada, and Australia has shown that medical students and doctors from black and minority ethnic (BME) backgrounds and/or doctors who obtained their primary medical qualification (PMQ) outside of the country in which they are practising do not perform as well as their colleagues who are white or trained in the country they practise in across a range of undergraduate and postgraduate medical assessments [9–17]. There is also evidence to suggest that male doctors who are or have been registered to practise medicine in the UK are more likely to have qualified in medicine outside of the UK [7].

The current study focuses on sex differences in performance on the Membership of the Royal Colleges of Physicians (UK) Practical Assessment of Clinical Examinations Skills (PACES), a standardised clinical examination in internal medicine taken by around 6000 candidates annually in the United Kingdom (UK) and 14 other countries worldwide. A previous study found female PACES candidates in 2003-4 performed statistically significantly better than male candidates after controlling for ethnicity [9]; however this population consisted only of candidates who had graduated in the UK precluding the possibility of exploring the interaction with country of qualification and country of sitting. In addition, that study used data from a previous format of the PACES examination: it has changed considerably since then [18].

We aimed to establish whether sex differences in performance were present in the new format PACES, and whether any sex differences varied by candidates’ country of PMQ, whether or not they were registered with the UK General Medical Council (GMC) and therefore likely to be working in the UK, and whether or not they sat the examination in the UK.

## Methods

### Study design, setting and source of data

This cross-sectional study was conducted using an international database from the Federation of Royal Colleges of Physicians in the UK, which organises the MRCP (UK) internal medicine specialty exams. The data and permission to use the data for research purposes were obtained from MRCP (UK).

### Membership of the Royal Colleges of physicians (UK) diploma

In the UK, doctors who wish to enter into higher specialist training in internal medicine are required to complete a three-part examination known as the Membership of the Royal College of Physicians United Kingdom (MRCP (UK)) Diploma, which aims to test the knowledge, skills, and behaviour of doctors in training [19]. The MRCP (UK) Diploma consists of three parts: MRCP (UK) Part 1; MRCP (UK) Part 2 Written; and MRCP (UK) Part 2 Practical Assessment of Clinical Examination Skills (PACES). Candidates are required to successfully complete all three parts of the exam before they are able to start specialist internal medicine training in the UK [19].

Doctors in many countries outside the UK sit the examination. In some countries (e.g. India) the MRCP (UK) qualification has status similar to local graduate training programmes. In others (e.g. Hong Kong) it forms part of a conjoint qualification. In countries where doctors may have difficulty accessing any formal training programmes, the MRCP (UK) qualification is used to benchmark a doctor’s knowledge and clinical skills against an internationally recognised standard. Completion of the MRCP (UK) diploma can improve a doctor’s chances of getting GMC registration and a license to practise in the UK, but anecdotal evidence suggests the majority of doctors sitting internationally do not come to the UK to work. MRCP (UK) does not collect employment status from candidates in the UK or internationally. It records registration with the GMC but not with any other regulatory bodies. GMC registration can be used as a proxy for current or previous employment in the UK.

**Fig. 1:**
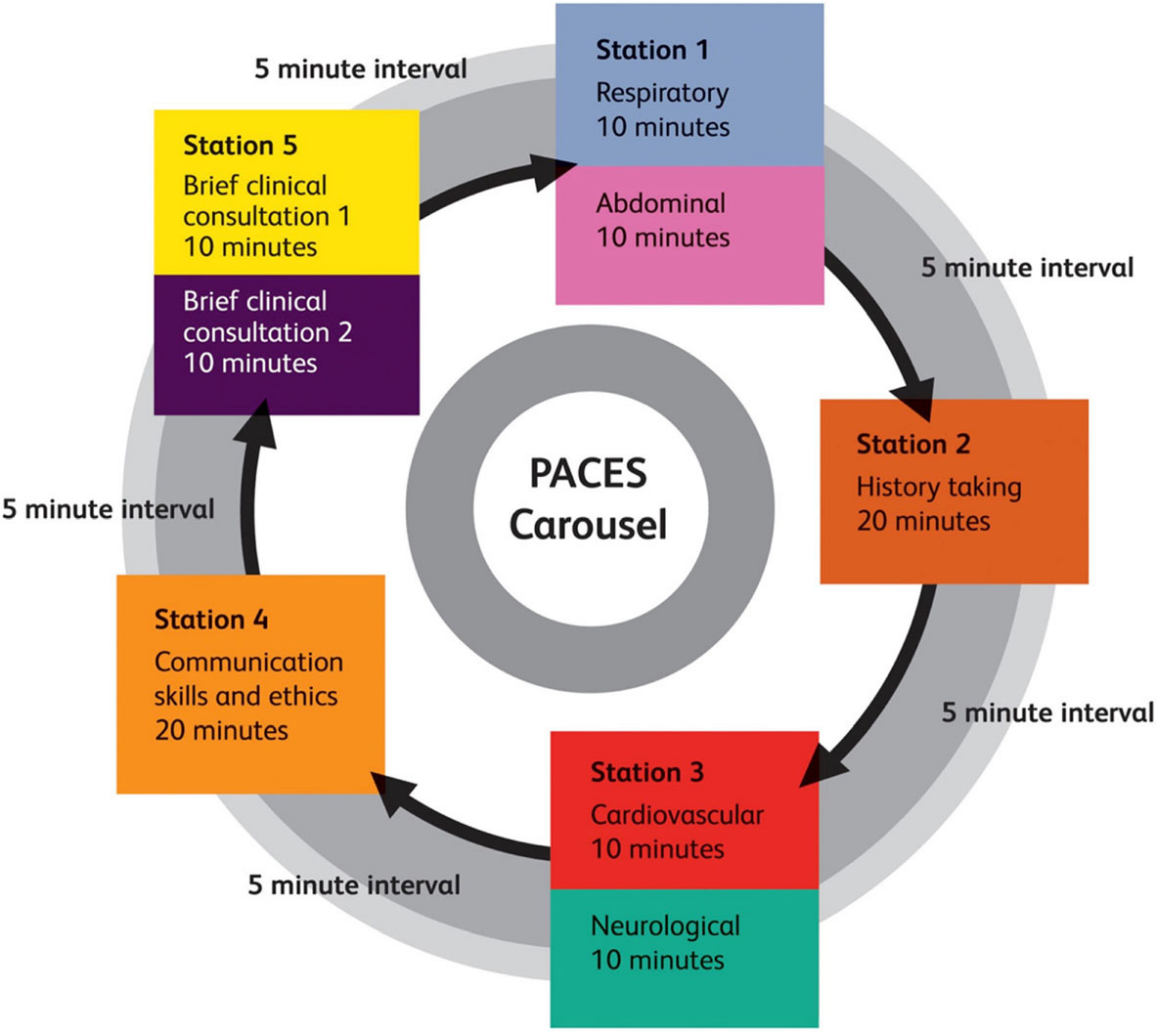
Carousel of PACES stations (source: MRCP (UK), permission obtained to reproduce figure in September 2016)

The current study focuses on the performance of candidates at the clinical assessment component of the Diploma, PACES. PACES is a structured standardised assessment that was first introduced in 2001, with the aim of providing a valid and reliable assessment of physical examination and communication skills [20]. PACES is run at clinical centres across the UK and in 14 other countries [21]. In 2009, the format of the exam was revised considerably with an aim of ensuring that successful candidates were competent across the range of clinical skills assessed, and that they possessed the attitudes and behaviour required of a specialist trainee in internal medicine [18]. Following a transitional phase between October 2009 and July 2010, the new format of the PACES assessment was introduced for all candidates from October 2010 [18]. Today PACES consists of five stations and a total of eight patient encounters, during which seven core clinical skills relating to communication, physical examination and diagnostic reasoning are assessed (see Fig. 1). Each station has two examiners who independently judge the candidate’s performance (10 examiners per PACES examination). Different skills are assessed at different stations i.e. not all stations test all skills. At each station, each examiner scores the relevant skills as 2 = satisfactory, 1 = borderline, 0 = unsatisfactory. A candidate must achieve a passing score in each skill to pass the examination overall. See https://www.mrcpuk.org/mrcpuk-examinations/paces/paces-examination-format for more details of the examination format and scoring.

PACES examiners must be registered with the General Medical Council (or regulatory equivalent in country of practice), be registered with a licence to practise, and be in good standing. They must also be a Collegiate Member or Fellow in good standing of one of the Royal College of Physicians of the UK. Collegiate Members must have achieved the Certificate of Completion of Training (CCT), or be on the specialist register, and be in a substantive consultant post. Physicians who are resident outside the UK and who wish to examine must hold Fellowship of one of the UK colleges of physicians and be in good standing. When the examination is taken outside of the UK, one of the examiners must be from the UK, and one must be from the host country. New examiners complete 3 days of training, and once trained, they must commit to examine at least six cycles (30 candidates) per annum averaged over 2 years. If they cannot do that, they must refresh their examiner training.

### Population and primary outcome

The study population included all doctors attempting PACES for the first time, at any of the official MRCP (UK) examination centres in the UK or internationally between October 2010 and May 2013. The pass standard did not change over the study period. We chose to restrict the study population to those attempting PACES for the first time, first attempt score being good predictor of score at subsequent attempts [22]. The predictor variable of interest was the candidates’ sex, as declared by the candidate to MRCP (UK).

### Selection of variables

The selection of co-variates was constrained data routinely collected by MRCP (UK). Co-variates were selected prior to any statistical analysis and selection was based on published findings that suggested they may influence and confound the association between candidates’ sex and performance at examinations [7, 9–13]. Variables with multiple categories were collapsed to create meaningful binary categories in order to increase statistical power. Included covariates were: ethnicity (white vs BME); country of PMQ (UK vs non-UK); GMC registration (registered vs not registered); country of PACES centre (UK vs non-UK).

### Statistical methods

We first performed bivariate analyses, examining the association between candidates’ sex and the other categorical variables; and between PACES pass rates and other categorical variables. Then we completed multivariate analyses using binary logistic regression models to examine the association between candidates’ sex and PACES pass rates, controlling for the other variables.

### Subgroup analyses

Our initial logistic regression model included candidates’ sex and pass rates, ethnicity, PMQ, GMC registration, and PACES examination centre location. Given the correlations between these variables and the risk of multicollinearity, we then decided to perform subgroup analyses to remove any possible correlation from the models. We divided the study population into three groups, representing the three broad groups of candidates who choose to complete PACES:

i) Candidates who had obtained their PMQ from a university in the UK (UK medical graduates). These doctors are predominantly working in UK training posts.
ii) Candidates who obtained their PMQ from an institution outside of the UK (non-UK medical graduates), and who were registered with the GMC. This population is likely to represent doctors working as doctors in the UK some of whom will be in training posts. These doctors are likely to have had clinical experience both abroad and in the UK.
iii) Candidates who obtained their PMQ from an institution outside of the UK (non-UK medical graduates), and who were not registered with the GMC. This population of doctors are currently unable to practise in the UK, and are therefore likely to have had the majority of their clinical experience, employment and training outside the UK.

Statistical analyses were conducted using the software Stata V.12/SE.

The study followed guidelines set out by the STROBE statement [23].

## Results

### Descriptive analyses

Seven thousand six hundred seventy-one candidates attempted PACES for the first time between October 2010 and May 2013. One candidate was excluded from all further analyses because they did not declare their sex. Of the remaining candidates 53% were men; 54% were from a black and minority ethnicity (BME) background (11% missing ethnicity data); 52% were UK medical graduates (< 1% missing PMQ data); 66% were registered with the GMC to practise in the UK (0% missing GMC registration data); and 77% sat PACES in a UK-based examination centre (0% missing PACES centre data); see Table 1.

**Table 1:** Distribution of variables by sex of the candidates (*N* = 7670)

Variable	Male *N* = 4026 (% of males)	Female *N* = 3644 (% of females)	Statistical significance
Passed PACES at first attempt			
Yes	1681 (42)	2055 (56)	*χ*^*2*^(1) = 144, *P* < 0.001
No	2345 (58)	1589 (44)	
Ethnicity			
White	1101 (27)	1602 (44)	*χ*^*2*^(1) = 241, *P* < 0.001
BME	2466 (61)	1653 (45)	
Missing	459 (11)	389 (11)	
World region where Primary Medical Qualification received			
UK	1692 (42)	2254 (62)	*χ*^*2*^(1) = 302, *P* < 0.001
Outside of UK	2332 (58)	1390 (38)	
Missing	2 (< 1)	0 (0)	
Registered with the General Medical Council			
Yes	2352 (58)	2725 (75)	*χ*^*2*^(1) = 229, *P* < 0.001
No	1674 (42)	919 (25)	
Country of examination centre			
UK	2862 (71)	3004 (77)	*χ*^*2*^(1) = 137, *P* < 0.001
Outside of UK	1164 (29)	640 (18)	

### Bivariate associations between candidate sex and other variables

Table 1 shows the distribution of each variable by sex of the candidates. Male candidates were statistically significantly more likely to be from a BME background, more likely to be a non-UK medical graduate, less likely to be registered with the GMC, and more likely to sit PACES at a non-UK centre (all *P* < 0.001).

### Bivariate associations between PACES pass rates and other variables

For the remainder of the descriptive analyses, candidates with one or more variable missing were excluded, leaving a total of 6820 candidates. Of those, just under half (49%) passed PACES at their first attempt. Passing candidates were statistically more likely to be female rather than male [56% vs. 42%; *χ*^*2*^(1) = 144, *P* < 0.001]; white rather than BME [65% vs. 38%; *χ*^*2*^(1) = 463, *P* < 0.001]; be a UK rather than a non-UK medical graduate [64% vs 32%; *χ*^*2*^(1) = 695, *P* < 0.001]; to be registered with the GMC rather than not [55% vs. 35%; *χ*^*2*^(1) = 232, *P* < 0.001]; and to have completed the examination at a UK examination centre rather than a centre outside the UK [51% vs. 40%; *χ*^*2*^(1) = 65, *P* < 0.001].

In summary, ethnicity, PMQ world region, GMC registration, and location of the examination centre, were associated with passing PACES at first attempt and candidate sex. We therefore considered these four variables as confounders of the association between sex and passing PACES at first attempt.

### Candidates with missing data

We compared the PACES performance of the 851 candidates (11% of the study population) who were missing data for at least one variable, to the PACES performance of the 6820 candidates with no missing data. We found no evidence of a statistically significant difference between these two groups in terms of the outcome of interest (*P* = 0.6). Given these findings we felt that candidates with missing data could be removed from the regression analyses.

### Regression analyses

#### Initial logistic regression model (full study population)

Adjusting for the other variables, female candidates were significantly more likely to pass PACES at first attempt compared with the male candidates (OR = 1.43; 95% CI = 1.29-1.59; *P* < 0.0001).

#### Subgroup regression analyses

##### UK medical graduates (*N* = 3574)

Just over half of the study population were UK medical graduates (52%), of which the majority were women (58%). The majority were of white ethnicity (67%), 0.5% were not registered with the GMC, and 1% attempted PACES at an examination centre outside of the UK. Bivariate analyses demonstrated that the latter two co-variates were not statistically significantly associated with PACES pass rate or sex, and they were therefore removed from further analyses in this group.

Female UK graduates had 1.18 times the odds of passing PACES at first attempt compared with male UK graduates, adjusting for ethnicity (OR = 1.18; 95% CI = 1.03-1.35; *P* = 0.02); see Table 2. There was no evidence of an interaction between candidate sex and ethnicity (*P* = 0.38), nor was there evidence of multicollinearity.

**Table 2:** The adjusted odds ratio (OR) for passing PACES at first attempt for female candidates compared to male candidates, and black and minority ethnic (BME) candidates compared to white candidates, after adjusting for all other variables (see text for details)

Variable	Adjusted OR	95% CI	*P*-value
UK graduates (*N* = 3574)			
Female	1.18	1.03-1.35	0.02
BME	0.60	0.52-0.69	< 0.0001
Non-UK graduates registered with the GMC (*N* = 1067)			
Female	1.47	1.11-1.94	< 0.01
BME	0.66	0.47-0.91	0.01
Non-UK graduates not registered with the GMC (*N* = 2179)			
Female	1.99	1.65-2.39	< 0.0001
BME	0.53	0.35-0.80	< 0.0001

Separate analyses performed for UK graduates, non-UK graduates registered with the General Medical Council (GMC), and non-UK graduates not registered with the GMC

##### Non-UK medical graduates registered with the GMC (*N* = 1067)

Sixteen percent of the study population had received their PMQ outside of the UK and were registered with the GMC to practice in the UK, of which men formed the majority (58%). The majority of this group declared themselves to be of BME background (81%), and only 38 candidates had completed PACES in an examination centre outside of the UK (4%). Bivariate analyses showed that whether the candidate had completed PACES in an examination centre based in the UK was not associated with PACES pass rate or with sex, and it was removed from further analyses in this group.

Female non-UK graduates registered with the GMC had nearly one and a half times the odds of passing PACES compared to their male counterparts (OR = 1.47; 95% CI = 1.11-1.94; *P* < 0.01); see Table 2. There was no evidence of an interaction between candidates’ sex and ethnicity (*P* = 0.17), nor was there evidence of any multicollinearity.

##### Non-UK medical graduates not registered with the GMC (*N* = 2179)

Nearly one third of the study population had received their PMQ from an institution outside of the UK, and were not registered with the GMC at the time of completing PACES (32%). Two thirds of this group was male (66%), and the vast majority reported themselves to be from a BME background (95%). The majority completed PACES at an examination centre outside of the UK (65%), it is likely that the remainder travelling to the UK solely to sit the examination. Bivariate analyses demonstrated that candidates’ ethnicity was associated PACES pass rate and sex. Centre location was found to be associated with PACES pass rate but not sex, therefore we chose not to include it in the regression model.

Female non-UK graduates who were not registered with the GMC had nearly twice the odds of passing PACES compared to their male counterparts (OR = 1.99; 95% CI = 1.65-2.39; *P* < 0.0001); see Table 2. There was no evidence of an interaction between candidates’ sex and ethnicity (*P* = 0.72), nor was there evidence of any multicollinearity.

## Discussion

This large cross-sectional study of doctors attempting the largest high stakes postgraduate clinical examination in the world [24] has demonstrated that female candidates are more likely to pass the examination at first attempt, even when adjusting for the candidates’ ethnicity. Ethnicity has been shown to influence candidates’ performance in examinations [10, 12, 13, 25], and therefore it is important to control for its effect. The size of the sex effect differed between the three groups examined, the largest being in non-UK medical graduates not registered with the GMC, the smallest in UK graduates registered with the GMC, and an intermediate effect size in non-UK medical graduates registered with the GMC.

### Comparison with other studies

The finding that female doctors are more likely to pass PACES mirrors the findings of Dewhurst and colleagues [9], who analysed data from the pre-2009 version of PACES in UK graduates only. They found that, after controlling for ethnicity, women had 1.69 the odds of passing (95% CI = 1.42–2.02), a significantly larger order of magnitude to the finding from our logistic regression examining UK graduates only (OR = 1.18; 95% CI = 1.03-1.35).

Sex differences in performance at clinical assessments in the UK in other specialties has also been shown, with women performing better than men at the General Practice specialty examination in both the written Applied Knowledge Test and the practical Clinical Skills Assessment [10, 26]. Women also performed better in the intercollegiate specialty board examinations for surgical training [27]. Indeed, a recent unpublished meta-analysis of UK-based studies has also demonstrated that female doctors perform better than male doctor at postgraduate medical examinations of a clinical nature, although sex differences are generally less pronounced in written examinations [28]. Interestingly the effect of sex is not so clear outside of the UK. In the US, the USMLE Step 3 is the final and clinical component of the medical licensing examination. Two studies have examined the sex difference in performance at this examination in US medical graduates, with one study finding women outperforming men [29], but the other study demonstrated no sex difference [30].

Our finding that female international medical graduates outperform male international medical graduates is also seen in other UK and US postgraduate examinations [26, 31–33]. To our knowledge, there are no studies looking at sex differences in the examination performance of international medical graduates with and without registration to practise medicine in the country in which the examination is set.

### Limitations of the study

The data were collected for routine administrative purposes, which limited our ability to gather potentially relevant data such as doctor’s age; however this also meant that the data on many variables were complete. The variable that contributed to the majority of the missing data was ethnicity, which was self-declared; however, a comparison of candidates with and without missing data showed no evidence of a difference in terms of passing PACES.

### Unanswered questions

It is not clear why candidate demographics relate to PACES outcome. Female doctors may be better at performing the skills tested in clinical assessments [28, 32]. It has been demonstrated that, during one clinical assessment, women ask more relevant history taking items and perform correctly more physical examination manoeuvres [33]. It could be that women are better at retaining and appropriately applying theoretical scientific and medical knowledge to clinical encounters in examinations; although scientific theoretical medical knowledge is formally assessed through written assessments [34] and a recent unpublished meta-analysis showed that sex differences were smaller or not present on written assessments. Further, there was no evidence for a sex difference in the written components of the MRCP (UK) Diploma when examined by Dewhurst and colleagues in 2003/04 [9], although it would be of interest to analyse sex difference on the written MRCP (UK) components during the time of period of the current study.

A popular hypothesis is that sex differences in performance are due to differences in communication styles. A meta-analytic review of medical consultations found that female doctors are more likely to adopt a patient-centred communication style [35]. It has also been demonstrated that women doctors have greater interpersonal skills, which lead to empathic relationships [36–38]. These interpersonal skills may encourage the patient to be more forthcoming with regards to salient clinical information, enabling the doctor to correctly diagnose and manage the presenting ailment. However, this hypothesis would not completely explain the sex difference seen in performance at PACES, because there are stations where there is no meaningful verbal interaction with the patient.

A further hypothesis is that male and female doctors differ in values, and that these values lead to different motivations, which in turn influence achievement. Female doctors have been shown to have higher person-related values [37, 39, 40], and one study found that performance in a clinical setting was predicted by person-related values held by the doctor [39]. It could also be that the design of the exam favours female candidates, perhaps examiners of clinical assessments may be unfairly biased towards female candidates or against male candidates. However there is no evidence to suggest a sex bias in clinical examiners in this current format of the PACES examination when assessed between 2009 and 2011 [41].

We did not examine the individual countries from which candidates had obtained their PMQ, but it is possible that the female candidates were more likely to have graduated from English-speaking countries when compared to male international medical graduates. Native English speakers perform better at clinical assessments conducted in English [10], and if women international medical graduates are found to be more likely from a country where English is the dominant language, or where communication skills and cultural values are more similar to the UK when compared to men, this could go toward explaining the sex difference in performance seen in international medical graduates. It may also reflect differences in the selection and training of female doctors in countries around the world [42]. It is also possible that access to PACES and medical education in general may be biased outside of the UK. This may plausibly result in female candidates needing to be higher performers and to be more motivated than their male counterparts, to overcome obstacles that may limit their access to the examination.

Candidates’ age was not examined in this study, but it is likely that non-UK graduates were older and a previous study has demonstrated that older candidates do not perform as well as younger candidates in clinical assessments [33]. The variation between the sex difference in non-UK graduates with and without GMC registration may reflect gendered migration patterns. For example, Lebanese medical graduates practising in the US are significantly less likely to be female than graduates of other countries [43], and a study of Lebanese medical students by the same authors found that female students had less intention of working abroad after graduation than male students [44]. It would be of interest to explore whether the difference in PACES performance has varied year upon year, or whether the sex difference is stable. This study captures just under 3 years worth of data and therefore it is unlikely that any meaningful conclusions will able to be drawn with regards to performance over time.

It is likely that performance in large, high-stakes clinical examinations that have demonstrated good validity reflects performance in actual clinical practice. A study comparing the MRCP (UK) scores of doctors who had and had not had their registration subject to action by the GMC found that those whose license had been acted upon had lower PACES scores [45]. It may be that the factors underlying sex differences in performance in clinical examinations also contribute to female doctors being less subject to medico-legal action [6].

## Conclusions

Female doctors outperform their male counterparts in a high stakes clinical exam once adjusted for the effect of their ethnicity, and the size of this sex effect was greater in doctors who graduated outside of the UK, especially those who were not registered with the UK’s GMC. Further work is required to understand the clinical significance of academic sex differences and the reasons sex differences in PACES performance vary by country.

## Abbreviations

95% CI: 95% confidence interval
BME: Black and Minority Ethnicity
GMC: General Medical Council
MRCP: Membership of the Royal College of Physicians
OR: Odds ratio
PACES: Practical Assessment of Clinical Examination Skills
PMQ: Primary Medical Qualification
UK: United Kingdom
US: United States
